# Supplementary material for: Multimorbidity and adverse events of special interest associated with Covid-19 vaccines in Hong Kong
Source: Nat Commun. 2022 Jan 20;13:411. doi: 10.1038/s41467-022-28068-3 (PMC8776841; doi:10.1038/s41467-022-28068-3)
Supplement: Supplementary file 1 — Supplementary Information [file 41467_2022_28068_MOESM1_ESM.pdf]

Manuscript Title: *Multimorbidity and adverse events of special interest associated with Covid-19 vaccines in Hong Kong*
Journal: Nature Communications

Supplementary Table 1 Frequency table of the multimorbidity status by age and vaccine groups

	Number of chronic conditions						
Age group	One	Two	Three	Four	Five	Six	Seven
Unvaccinated (n=547796)							
16-19	2644 (0.77)	128 (0.08)	6 (0.02)	0 (0)	0 (0)	0 (0)	0 (0)
20-24	4648 (1.35)	289 (0.17)	14 (0.05)	0 (0)	0 (0)	0 (0)	0 (0)
25-29	6192 (1.80)	448 (0.27)	39 (0.14)	3 (0.06)	0 (0)	0 (0)	0 (0)
30-34	8190 (2.37)	811 (0.48)	73 (0.25)	6 (0.12)	0 (0)	0 (0)	0 (0)
35-39	10735 (3.11)	1555 (0.92)	146 (0.51)	18 (0.36)	1 (0.12)	0 (0)	1 (10)
40-44	14152 (4.10)	2922 (1.74)	331 (1.15)	48 (0.95)	4 (0.48)	0 (0)	0 (0)
45-49	20760 (6.02)	5673 (3.37)	694 (2.41)	82 (1.63)	20 (2.42)	2 (1.69)	0 (0)
50-54	28704 (8.32)	9979 (5.93)	1374 (4.78)	185 (3.67)	21 (2.54)	2 (1.69)	0 (0)
55-59	44051 (12.77)	19184 (11.41)	2788 (9.69)	396 (7.85)	67 (8.1)	5 (4.24)	0 (0)
60-64	58911 (17.08)	30468 (18.12)	4668 (16.23)	739 (14.66)	122 (14.75)	23 (19.49)	1 (10)
65-69	60866 (17.65)	35966 (21.39)	6094 (21.18)	1067 (21.16)	186 (22.49)	23 (19.49)	1 (10)
70-74	49221 (14.27)	33614 (19.99)	6261 (21.76)	1247 (24.73)	212 (25.63)	36 (30.51)	4 (40)
75-79	21585 (6.26)	16572 (9.85)	3658 (12.72)	714 (14.16)	113 (13.66)	14 (11.86)	0 (0)
80-84	9450 (2.74)	7279 (4.33)	1739 (6.05)	348 (6.9)	53 (6.41)	12 (10.17)	1 (10)
85+	4757 (1.38)	3278 (1.95)	882 (3.07)	189 (3.75)	28 (3.39)	1 (0.85)	2 (20)
Comirnaty (n=153178)							
16-19	1352 (1.18)	45 (0.13)	0 (0)	0 (0)	0 (0)	0 (0)	0 (0)
20-24	2394 (2.09)	113 (0.33)	8 (0.19)	0 (0)	0 (0)	0 (0)	0 (0)
25-29	2906 (2.54)	182 (0.53)	11 (0.26)	1 (0.19)	0 (0)	0 (0)	0 (0)
30-34	3693 (3.23)	334 (0.98)	13 (0.31)	2 (0.39)	0 (0)	0 (0)	0 (0)
35-39	5309 (4.64)	561 (1.65)	39 (0.92)	3 (0.58)	1 (1.96)	0 (0)	0 (0)
40-44	7679 (6.72)	1110 (3.26)	76 (1.8)	7 (1.35)	0 (0)	0 (0)	0 (0)
45-49	10781 (9.43)	2098 (6.16)	205 (4.85)	15 (2.9)	2 (3.92)	0 (0)	0 (0)
50-54	14126 (12.36)	3471 (10.19)	372 (8.81)	41 (7.93)	0 (0)	1 (33.33)	0 (0)
55-59	18537 (16.22)	5703 (16.74)	588 (13.92)	60 (11.61)	4 (7.84)	1 (33.33)	0 (0)
60-64	18520 (16.2)	6784 (19.91)	789 (18.68)	89 (17.21)	16 (31.37)	0 (0)	1 (100)
65-69	14057 (12.3)	5720 (16.79)	808 (19.13)	103 (19.92)	11 (21.57)	1 (33.33)	0 (0)
70-74	9192 (8.04)	4643 (13.63)	669 (15.84)	93 (17.99)	16 (31.37)	0 (0)	0 (0)
75-79	3587 (3.14)	2013 (5.91)	387 (9.16)	56 (10.83)	1 (1.96)	0 (0)	0 (0)
80-84	1495 (1.31)	881 (2.59)	176 (4.17)	28 (5.42)	0 (0)	0 (0)	0 (0)
85+	683 (0.6)	413 (1.21)	83 (1.96)	19 (3.68)	0 (0)	0 (0)	0 (0)
CoronaVac (n=182442)							
16-19	160 (0.12)	12 (0.03)	1 (0.02)	0 (0)	0 (0)	0 (0)	0 (0)
20-24	506 (0.39)	28 (0.06)	2 (0.03)	0 (0)	0 (0)	0 (0)	0 (0)
25-29	721 (0.56)	37 (0.08)	3 (0.05)	0 (0)	0 (0)	0 (0)	0 (0)
30-34	1328 (1.03)	101 (0.22)	5 (0.08)	1 (0.14)	0 (0)	0 (0)	0 (0)
35-39	2700 (2.09)	288 (0.61)	20 (0.34)	0 (0)	0 (0)	0 (0)	0 (0)
40-44	5273 (4.09)	741 (1.58)	59 (1)	3 (0.42)	1 (1.16)	0 (0)	0 (0)
45-49	10127 (7.86)	1850 (3.95)	150 (2.55)	15 (2.09)	2 (2.33)	0 (0)	0 (0)

50-54	15280 (11.86)	3743 (7.99)	346 (5.87)	36 (5.01)	1 (1.16)	1 (9.09)	0 (0)
55-59	21731 (16.86)	7065 (15.08)	714 (12.12)	72 (10.03)	10 (11.63)	0 (0)	0 (0)
60-64	23995 (18.62)	9378 (20.02)	1047 (17.77)	118 (16.43)	8 (9.3)	5 (45.45)	0 (0)
65-69	21066 (16.34)	9249 (19.74)	1206 (20.46)	155 (21.59)	16 (18.6)	2 (18.18)	0 (0)
70-74	15142 (11.75)	7831 (16.72)	1193 (20.24)	142 (19.78)	23 (26.74)	2 (18.18)	0 (0)
75-79	6264 (4.86)	3827 (8.17)	619 (10.5)	87 (12.12)	14 (16.28)	1 (9.09)	0 (0)
80-84	3025 (2.35)	1820 (3.89)	329 (5.58)	63 (8.77)	7 (8.14)	0 (0)	0 (0)
85+	1572 (1.22)	874 (1.87)	199 (3.38)	26 (3.62)	4 (4.65)	0 (0)	0 (0)

Manuscript Title: *Multimorbidity and adverse events of special interest associated with Covid-19 vaccines in Hong Kong*
Journal: Nature Communications

Supplementary Table 2. Frequency table of chronic disease and specific adverse events of special interest by vaccine groups

	Adverse events of special interest							
Chronic diseases	Auto-immune	Cardiovascular	Circulatory	Hepato-renal	Nerves and central nervous	Respiratory	Skin, bone and joints	Others
Unvaccinated (n=547796)								
Alcohol misuse	3	7	6	9	4	3	0	3
Asthma	13	20	8	9	1	8	0	7
Cancer	19	42	47	31	15	42	1	13
Chronic pain	11	43	38	21	2	13	0	15
Chronic pulmonary disease	8	34	22	10	2	15	0	4
Cirrhosis	2	5	10	9	1	5	1	1
Dementia	2	8	14	2	2	3	0	3
Depression	21	44	31	24	5	9	0	9
Hypertension	161	569	538	390	30	89	0	67
Hypothyroidism	9	30	20	18	3	5	0	7
IBD	0	1	2	1	0	1	0	1
IBS	0	1	3	1	0	0	0	0
Parkinson's disease	1	6	6	0	0	0	0	2
Peptic ulcer disease	2	15	14	5	0	5	1	2
Peripheral vascular disease	0	0	0	0	0	0	0	0
Psoriasis	2	5	4	6	0	0	0	1
Rheumatoid arthritis	7	7	12	1	1	4	0	0
Schizophrenia	7	3	8	5	3	3	0	9
Severe constipation	23	93	76	51	7	23	0	13
Type 2 diabetes	65	299	259	230	21	43	0	44
Comirnaty (n=153178)								
Alcohol misuse	1	1	0	1	2	0	0	0
Asthma	2	3	5	2	0	0	1	3
Cancer	1	2	5	0	1	0	0	1
Chronic pain	0	3	3	5	2	1	0	3
Chronic pulmonary disease	1	0	1	1	0	3	0	0
Cirrhosis	0	1	1	0	0	0	0	0
Dementia	0	0	0	0	0	0	0	0
Depression	7	6	6	4	1	0	1	10
Hypertension	37	87	91	65	8	8	1	19
Hypothyroidism	3	7	3	6	1	3	0	1
IBD	0	0	0	1	0	0	0	0
IBS	0	0	0	0	0	0	0	0
Parkinson's disease	2	1	1	1	0	0	0	0
Peptic ulcer disease	0	0	0	0	1	0	0	0
Peripheral vascular disease	0	0	0	0	0	0	0	0
Psoriasis	1	0	0	0	0	0	1	2
Rheumatoid arthritis	0	1	2	1	0	0	0	1
Schizophrenia	0	2	0	1	1	0	0	0

Severe constipation	10	9	12	6	1	1	2	8
Type 2 diabetes	8	26	30	23	5	2	0	7
CoronaVac (n=182442)								
Alcohol misuse	2	0	1	1	0	0	0	1
Asthma	2	7	7	3	1	1	1	2
Cancer	1	2	1	2	0	0	0	1
Chronic pain	1	14	15	9	4	1	0	3
Chronic pulmonary disease	1	6	1	1	0	2	0	0
Cirrhosis	1	1	1	1	0	0	0	1
Dementia	0	0	0	0	0	0	0	0
Depression	5	9	5	6	1	2	0	4
Hypertension	26	120	106	114	11	12	0	29
Hypothyroidism	3	5	7	2	0	1	0	2
IBD	0	0	0	1	0	0	0	0
IBS	0	0	0	0	0	0	0	0
Parkinson's disease	0	2	0	0	0	0	0	0
Peptic ulcer disease	0	4	2	1	0	0	0	0
Peripheral vascular disease	0	0	0	0	0	0	0	0
Psoriasis	0	0	0	1	1	0	0	0
Rheumatoid arthritis	0	1	1	1	0	0	0	0
Schizophrenia	0	0	0	1	0	0	0	1
Severe constipation	6	15	13	10	1	1	0	2
Type 2 diabetes	15	53	47	62	3	6	0	14

IBS = Irritable bowel syndrome; IBD = Inflammatory bowel disease

Supplementary Table 3. Frequency of each specific adverse events of interest (%) within observation period by vaccine groups

Adverse events of special interest	Unvaccinated (n=547796)	Comirnaty (n=153178)	CoronaVac (n=182442)
Auto-immune diseases			
Guillain-Barre Syndrome	8 (0.0)	1 (0.0)	1 (0.0)
Acute disseminated encephalomyelitis (ADEM)	0 (0.0)	0 (0.0)	0 (0.0)
Narcolepsy	170 (0.03)	42 (0.03)	31 (0.02)
Acute aseptic arthritis	24 (0.0)	2 (0.0)	6 (0.0)
Type 1 Diabetes	4 (0.0)	0 (0.0)	0 (0.0)
(Idiopathic) Thrombocytopenia	22 (0.0)	0 (0.0)	2 (0.0)
Subacute thyroiditis	1 (0.0)	0 (0.0)	0 (0.0)
Cardiovascular system diseases			
Microangiopathy	1 (0.0)	0 (0.0)	0 (0.0)
Heart failure	77 (0.01)	3 (0.0)	9 (0.01)
Stress cardiomyopathy	0 (0.0)	0 (0.0)	0 (0.0)
Coronary artery disease	430 (0.08)	51 (0.03)	90 (0.05)
Arrhythmia	276 (0.05)	33 (0.02)	60 (0.03)
Carditis	17 (0.0)	1 (0.0)	3 (0.0)
Circulatory system diseases			
Thromboembolism	642 (0.12)	87 (0.06)	122 (0.07)
Haemorrhagic disease	183 (0.03)	17 (0.01)	30 (0.02)
Single Organ Cutaneous Vasculitis	3 (0.0)	3 (0.0)	4 (0.0)
Hepato-renal system diseases			
Acute liver injury	276 (0.05)	46 (0.03)	99 (0.05)
Acute kidney injury	223 (0.04)	17 (0.01)	46 (0.03)
Acute pancreatitis	16 (0.0)	3 (0.0)	1 (0.0)
Nerves and central nervous system			
Generalized convulsion	33 (0.01)	8 (0.01)	5 (0.0)
Meningoencephalitis	7 (0.0)	0 (0.0)	0 (0.0)
Transverse myelitis	0 (0.0)	0 (0.0)	0 (0.0)
Bell’s Palsy	24 (0.0)	4 (0.0)	9 (0.0)
Respiratory system disease			
Acute respiratory distress syndrome	154 (0.03)	7 (0.0)	18 (0.01)
Skin, bone, and joints system diseases			
Erythema multiforme	2 (0.0)	1 (0.0)	0 (0.0)
Chilblain – like lesions	0 (0.0)	1 (0.0)	1 (0.0)
Other system diseases			
Anosmia, ageusia	1 (0.0)	0 (0.0)	3 (0.0)
Anaphylaxis	98 (0.02)	29 (0.02)	36 (0.02)
Multisystem inflammatory Syndrome in children	0 (0.0)	0 (0.0)	0 (0.0)
Rhabdomyolysis	12 (0.0)	0 (0.0)	2 (0.0)

Supplementary Table 4. Hazard ratios with 95% confidence intervals (CI) of adverse events of interest generated from Cox proportional hazard models with entropy rebalancing (sub-categories of the AESI as outcomes)

	Hazard ratios with 95% CI, P-value		
	Model 1	Model 2	Model 3
Auto-immune diseases			
Vaccination status			
Unvaccinated	Ref	Ref	Ref
Comirnaty	0.84 (0.58, 1.19), 0.321	0.84 (0.59, 1.19), 0.327	0.82 (0.54, 1.24), 0.343
CoronaVac	0.55 (0.39, 0.79), 0.001	0.55 (0.39, 0.79), 0.001	0.49 (0.31, 0.77), 0.002
2Multimorbidity status			
One chronic condition	-	Ref	Ref
Multimorbid	-	1.22 (0.96, 1.57), 0.100	1.16 (0.88, 1.55), 0.294
Interaction			
Comirnaty X multimorbidity	-	-	1.08 (0.52, 2.24), 0.835
CoronaVac X multimorbidity	-	-	1.36 (0.65, 2.85), 0.408
Cardiovascular system diseases			
Vaccination status			
Unvaccinated	Ref	Ref	Ref
Comirnaty	0.58 (0.45, 0.75), <0.001	0.59 (0.45, 0.76), <0.001	0.59 (0.42, 0.82), 0.002
CoronaVac	0.65 (0.54, 0.79), <0.001	0.66 (0.54, 0.80), <0.001	0.67 (0.53, 0.86), 0.002
Multimorbidity status			
One chronic condition	-	Ref	Ref
Multimorbid	-	1.66 (1.44, 1.90), <0.001	1.67 (1.43, 1.97), <0.001
Interaction			
Comirnaty X multimorbidity	-	-	0.99 (0.59, 1.66), 0.965
CoronaVac X multimorbidity	-	-	0.95 (0.64, 1.41), 0.793
Circulatory system diseases			
Vaccination status			
Unvaccinated	Ref	Ref	Ref
Comirnaty	0.77 (0.58, 1.01), 0.059	0.77 (0.59, 1.02), 0.065	0.82 (0.59, 1.15), 0.251
CoronaVac	0.58 (0.46, 0.73), <0.001	0.58 (0.46, 0.74), <0.001	0.50 (0.36, 0.69), <0.001
Multimorbidity status			
One chronic condition	-	Ref	Ref
Multimorbid	-	1.81 (1.54, 2.13), <0.001	1.77 (1.47, 2.12), <0.001
Interaction			
Comirnaty X multimorbidity	-	-	0.87 (0.49, 1.53), 0.629
CoronaVac X multimorbidity	-	-	1.36 (0.86, 2.16), 0.187
Hepato-renal system diseases			
Vaccination status			
Unvaccinated	Ref	Ref	Ref
Comirnaty	0.58 (0.44, 0.76), <0.001	0.58 (0.44, 0.77), <0.001	0.62 (0.44, 0.86), 0.005
CoronaVac	0.89 (0.74, 1.08), 0.235	0.90 (0.74, 1.08), 0.257	0.86 (0.67, 1.10), 0.223

Multimorbidity status			
One chronic condition	-	Ref	Ref
Multimorbid	-	1.54 (1.32, 1.79), <0.001	1.53 (1.28, 1.83), <0.001
Interaction			
Comirnaty X multimorbidity	-	-	0.87 (0.50, 1.54), 0.640
CoronaVac X multimorbidity	-	-	1.11 (0.75, 1.62), 0.610
Nerves and central nervous system diseases			
Vaccination status			
Unvaccinated	Ref	Ref	Ref
Comirnaty	0.73 (0.36, 1.46), 0.368	0.73 (0.36, 1.47), 0.375	0.91 (0.39, 2.14), 0.825
CoronaVac	0.71 (0.39, 1.28), 0.250	0.71 (0.39, 1.29), 0.258	0.76 (0.36, 1.61), 0.477
Multimorbidity status			
One chronic condition	-	Ref	Ref
Multimorbid	-	1.59 (1.03, 2.46), 0.038	1.74 (1.05, 2.91), 0.033
Interaction			
Comirnaty X multimorbidity	-	-	0.57 (0.13, 2.57), 0.466
CoronaVac X multimorbidity	-	-	0.86 (0.25, 2.89), 0.801
Respiratory system disease			
Vaccination status			
Unvaccinated	Ref	Ref	Ref
Comirnaty	0.21 (0.08, 0.61), 0.004	0.22 (0.08, 0.61), 0.004	0.21 (0.06, 0.81), 0.024
CoronaVac	0.31 (0.17, 0.57), <0.001	0.32 (0.17, 0.58), <0.001	0.56 (0.27, 1.14), 0.111
Multimorbidity status			
One chronic condition	-	Ref	Ref
Multimorbid	-	2.61 (1.85, 3.68), <0.001	2.93 (2.01, 4.24), 0.001
Interaction			
Comirnaty X multimorbidity	-	-	1.03 (0.14, 7.46), 0.975
CoronaVac X multimorbidity	-	-	0.27 (0.07, 1.08), 0.065
Skin and mucous membrane, bone and joints system disease			
Vaccination status			
Unvaccinated	Ref	Ref	Ref
Comirnaty	4.71 (0.25, 89.38), 0.302	4.74 (0.25, 98.87), 0.302	3.28 (0.15, 73.5), 0.454
CoronaVac	1.92 (0.17, 21.26), 0.596	1.93 (0.18, 21.13), 0.590	4.11 (0.26, 66.2), 0.318
Multimorbidity status			
One chronic condition	-	Ref	Ref
Multimorbid	-	1.65 (0.27, 10.22), 0.589	2.32 (0.01, 37.2), 0.551
Interaction			
Comirnaty X multimorbidity	-	-	1.84 (0.04, 94.1), 0.761
CoronaVac X multimorbidity	-	-	1.26e-08 (4.18e-10, 3.79e-07), <0.001
Other system diseases			
Vaccination status			
Unvaccinated	Ref	Ref	Ref

Comirnaty	0.97 (0.63, 1.50), 0.895	0.97 (0.63, 1.51), 0.905	1.13 (0.66, 1.94), 0.644
CoronaVac	1.24 (0.85, 1.79), 0.268	1.24 (0.85, 1.80), 0.260	1.27 (0.79, 2.05), 0.324
Multimorbidity status			
One chronic condition	-	Ref	Ref
Multimorbid	-	1.37 (1.01, 1.87), 0.046	1.47 (1.00, 2.17), 0.050
Interaction			
Comirnaty X multimorbidity	-	-	0.66 (0.26, 1.69), 0.391
CoronaVac X multimorbidity	-	-	0.94 (0.44, 2.03), 0.879

Included independent variable in Model 1: vaccination status only; Model 2: Model 1 + multimorbidity status; Model 3: Model 2 + interaction between vaccination status and multimorbidity

Supplementary Table 5. Hazard ratios with 95% confidence intervals (CI) of each specific adverse events of interest generated from Cox proportional hazard models with entropy rebalancing

Adverse events of special interest	Hazard ratios (95% CI), P-value				
	Comirnaty	CoronaVac	Multimorbid	Comirnaty X multimorbidity	CoronaVac X multimorbidity
Auto-immune diseases					
Narcolepsy	0.94 (0.61, 1.46), 0.798	0.47 (0.29, 0.78), 0.003	1.22 (0.90, 1.66), 0.206	0.97 (0.45, 2.08), 0.943	1.36 (0.61, 3.04), 0.450
Cardiovascular system diseases					
Heart failure	0.18 (0.03, 1.33), 0.093	0.25 (0.09, 0.69), 0.008	1.77 (1.13, 2.78), 0.013	2.08 (0.18, 24.00), 0.556	1.95 (0.48, 7.88), 0.347
Coronary artery disease	0.61 (0.42, 0.88), 0.008	0.58 (0.43, 0.78), <0.001	1.61 (1.33, 1.94), <0.001	0.90 (0.48, 1.70), 0.744	1.12 (0.70, 1.78), 0.641
Arrhythmia	0.66 (0.41, 1.07), 0.089	0.66 (0.46, 0.95), 0.026	1.67 (1.32, 2.11), <0.001	0.82 (0.38, 1.77), 0.617	1.14 (0.63, 2.04), 0.667
Circulatory system diseases					
Thromboembolism	0.75 (0.56, 1.00), 0.048	0.54 (0.42, 0.70), <0.001	1.62 (1.38, 1.89), <0.001	0.95 (0.58, 1.55), 0.823	1.12 (0.75, 1.66), 0.587
Haemorrhagic disease	0.40 (0.18, 0.88), 0.022	0.48 (0.29, 0.81), 0.006	1.67 (1.25, 2.23), 0.001	2.32 (0.80, 6.72), 0.121	1.07 (0.49, 2.33), 0.874
Single Organ Cutaneous Vasculitis	2.46 (0.22, 27.11), 0.463	2.80 (0.39, 19.90), 0.303	0.76 (0.07, 8.33), 0.819	4.70 (0.16, 139.77), 0.372	4.14 (0.18, 92.77), 0.370
Hepato-renal system diseases					
Acute liver injury	0.65 (0.44, 0.95), 0.027	0.95 (0.70, 1.28), 0.741	1.16 (0.91, 1.48), 0.218	0.89 (0.44, 1.79), 0.748	1.27 (0.79, 2.04), 0.316
Acute kidney injury	0.45 (0.22, 0.93), 0.031	0.63 (0.40, 0.97), 0.038	2.32 (1.78, 3.03), <0.001	0.94 (0.33, 2.67), 0.905	1.05 (0.54, 2.04), 0.882
Nerves and central nervous system					
Bell's Palsy	1.19 (0.32, 4.37), 0.792	0.82 (0.27, 2.53), 0.730	1.61 (0.72, 3.62), 0.248	0.30 (0.03, 3.34), 0.324	1.97 (0.42, 9.32), 0.392
Respiratory system disease					
Acute respiratory distress syndrome	0.21 (0.06, 0.81), 0.017	0.56 (0.27, 1.14), 0.014	2.93 (2.01, 4.24), <0.001	1.03 (0.14, 7.46), 0.988	0.27 (0.07, 1.08), 0.510
Other system diseases					
Anaphylaxis	1.27 (0.74, 2.18), 0.385	1.20 (0.72, 2.00), 0.494	1.54 (1.03, 2.30), 0.034	0.63 (0.25, 1.63), 0.342	1.06 (0.48, 2.34), 0.887

Included independent variable: vaccine groups + multimorbidity status + interaction between vaccination status and multimorbidity
Adverse event of special interest with any level of the independent variable containing fewer than 3 subjects were omitted from the analyses to avoid extreme estimates

Supplementary Table 6. Hazard ratios with 95% confidence intervals (CI) of adverse events of interest generated from Cox proportional hazard models with entropy rebalancing (multimorbidity status replaced by the Charlson Comorbidity Index score)

	Hazard ratios with 95% CI		
	Model 1	Model 2	Model 3
Vaccination status			
Unvaccinated	Ref	Ref	Ref
Comirnaty	0.64 (0.56, 0.73), <0.001	0.65 (0.57, 0.74), <0.001	0.77 (0.66, 0.90), <0.001
CoronaVac	0.68 (0.61, 0.75), <0.001	0.69 (0.62, 0.76), <0.001	0.69 (0.60, 0.80), <0.001
Multimorbidity status			
Charlson Comorbidity Index score	-	1.17 (1.13, 1.21), <0.001	1.19 (1.15, 1.23), <0.001
Interaction			
Comirnaty X Charlson Comorbidity Index score	-	-	0.72 (0.59, 0.88), 0.001
CoronaVac X Charlson Comorbidity Index score	-	-	0.99 (0.84, 1.16), 0.891

Included independent variable in Model 1: vaccination status only; Model 2: Model 1 + Charlson Comorbidity Index score; Model 3: Model 2 + interaction between vaccination status and Charlson Comorbidity Index score

Supplementary Table 7. Hazard ratios with 95% confidence intervals (CI) of adverse events of interest generated from Cox proportional hazard models with entropy rebalancing (date of second dose omitted as observation endpoints)

	Hazard ratios with 95% CI		
	Model 1	Model 2	Model 3
Vaccination status			
Unvaccinated	Ref	Ref	Ref
Comirnaty	0.70 (0.62, 0.79), <0.001	0.70 (0.63, 0.79), <0.001	0.76 (0.66, 0.88), <0.001
CoronaVac	0.70 (0.63, 0.78), <0.001	0.71 (0.64, 0.78), <0.001	0.70 (0.61, 0.80), <0.001
Multimorbidity status			
One chronic condition	-	Ref	Ref
Multimorbid	-	1.61 (1.49, 1.73), <0.001	1.64 (1.50, 1.79), <0.001
Interaction			
Comirnaty X multimorbidity	-	-	0.84 (0.66, 1.07), 0.161
CoronaVac X multimorbidity	-	-	1.02 (0.83, 1.25), 0.869

Included independent variable in Model 1: vaccination status only; Model 2: Model 1 + multimorbidity status; Model 3: Model 2 + interaction between vaccination status and multimorbidity

Supplementary Table 8. Hazard ratios with 95% confidence intervals (CI) of adverse events of interest generated from Cox proportional hazard models with entropy rebalancing (including only patients who were vaccinated on or before July 3, 2021)

	Hazard ratios with 95% CI		
	Model 1	Model 2	Model 3
Vaccination status			
Unvaccinated	Ref	Ref	Ref
Comirnaty	0.68 (0.58, 0.78), <0.001	0.68 (0.59, 0.79), <0.001	0.70 (0.58, 0.84), <0.001
CoronaVac	0.70 (0.63, 0.79), <0.001	0.71 (0.63, 0.79), <0.001	0.69 (0.60, 0.80), <0.001
Multimorbidity status			
One chronic condition	-	Ref	Ref
Multimorbid	-	1.64 (1.51, 1.79), <0.001	1.65 (1.50, 1.81), <0.001
Interaction			
Comirnaty X multimorbidity	-	-	0.93 (0.69, 1.25), 0.618
CoronaVac X multimorbidity	-	-	1.03 (0.82, 1.30), 0.779

Included independent variable in Model 1: vaccination status only; Model 2: Model 1 + multimorbidity status; Model 3: Model 2 + interaction between vaccination status and multimorbidity

Supplementary Table 9. Hazard ratios with 95% confidence intervals (CI) of adverse events of interest (AESI) generated from Cox proportional hazard models with entropy rebalancing (AESI defined by inpatient records only)

	Hazard ratios with 95% CI		
	Model 1	Model 2	Model 3
Vaccination status			
Unvaccinated	Ref	Ref	Ref
Comirnaty	0.58 (0.48, 0.70), <0.001	0.58 (0.48, 0.70), <0.001	0.66 (0.52, 0.84), <0.001
CoronaVac	0.52 (0.44, 0.62), <0.001	0.52 (0.44, 0.62), <0.001	0.53 (0.43, 0.66), <0.001
Multimorbidity status			
One chronic condition	-	Ref	Ref
Multimorbid	-	1.76 (1.57, 1.97), <0.001	1.82 (1.61, 2.06), <0.001
Interaction			
Comirnaty X multimorbidity	-	-	0.75 (0.50, 1.12), 0.165
CoronaVac X multimorbidity	-	-	0.98 (0.69, 1.38), 0.896

Included independent variable in Model 1: vaccination status only; Model 2: Model 1 + multimorbidity status; Model 3: Model 2 + interaction between vaccination status and multimorbidity

Supplementary Table 10. Hazard ratios with 95% confidence intervals (CI) of adverse events of interest generated from Cox proportional hazard models with entropy rebalancing (excluding patients with a hospitalization record in the past six months)

	Hazard ratios with 95% CI		
	Model 1	Model 2	Model 3
Vaccination status			
Unvaccinated	Ref	Ref	Ref
Comirnaty	0.77 (0.67, 0.89), <0.001	0.77 (0.67, 0.89), <0.001	0.80 (0.67, 0.95), 0.011
CoronaVac	0.87 (0.78, 0.97), 0.011	0.87 (0.78, 0.97), 0.012	0.81 (0.70, 0.93), 0.004
Multimorbidity status			
One chronic condition	-	Ref	Ref
Multimorbid	-	1.32 (1.21, 1.44), <0.001	1.30 (1.17, 1.44), <0.001
Interaction			
Comirnaty X multimorbidity	-	-	0.91 (0.68 1.23), 0.542
CoronaVac X multimorbidity	-	-	1.18 (0.94, 1.47), 0.158

Included independent variable in Model 1: vaccination status only; Model 2: Model 1 + multimorbidity status; Model 3: Model 2 + interaction between vaccination status and multimorbidity

Supplementary Table 11. Hazard ratios with 95% confidence intervals (CI) of adverse events of interest generated from Cox proportional hazard models with entropy rebalancing (the number of chronic conditions as effect modifier)

	Hazard ratios (95% CI)		
	Model 1	Model 2	Model 3
Vaccine groups			
Unvaccinated	Ref	Ref	Ref
Comirnaty	0.66 (0.58, 0.75), <0.001	0.66 (0.58, 0.75), <0.001	0.80 (0.59, 1.08), 0.144
CoronaVac	0.70 (0.63, 0.77), <0.001	0.70 (0.63, 0.78), <0.001	0.67 (0.52, 0.86), 0.002
Multimorbidity status			
Number of multimorbid	-	1.48 (1.41, 1.55), <0.001	1.49 (1.41, 1.57), <0.001
Interaction			
Comirnaty X multimorbidity	-	-	0.88 (0.73, 1.07), 0.212
CoronaVac X multimorbidity	-	-	1.03 (0.88, 1.20), 0.728

Included independent variable in Model 1: vaccine groups only; Model 2: Model 1 + multimorbidity status; Model 3: Model 2 + interaction between vaccination status and multimorbidity

Supplementary Table 12. Incidence rate ratio with 95% confidence intervals (CI) of adverse events of interest generated from Poisson regression models with entropy rebalancing

	Incidence rate ratios (95% CI)		
	Model 1	Model 2	Model 3
Vaccine groups			
Unvaccinated	Ref	Ref	Ref
Comirnaty	0.61 (0.54, 0.69), <0.001	0.67 (0.59, 0.75), <0.001	0.71 (0.60, 0.83), <0.001
CoronaVac	0.68 (0.61, 0.75), <0.001	0.70 (0.63, 0.78), <0.001	0.69 (0.60, 0.79), <0.001
Multimorbidity status			
One chronic condition	-	Ref	Ref
Multimorbid		1.63 (1.51, 1.75), <0.001	1.64 (1.50, 1.79), <0.001
Interaction			
Comirnaty X multimorbidity	-	-	0.88 (0.69, 1.12), 0.294
CoronaVac X multimorbidity	-	-	1.03 (0.84, 1.26), 0.753

Included independent variable in Model 1: vaccine groups only; Model 2: Model 1 + multimorbidity status; Model 3: Model 2 + interaction between vaccination status and multimorbidity

Supplementary Table 13. Hazard ratios with 95% confidence intervals (CI) of adverse events of interest generated from Cox proportional hazard models with propensity score weighting

	Hazard ratios (95% CI)		
	Model 1	Model 2	Model 3
Vaccine groups			
Unvaccinated	Ref	Ref	Ref
Comirnaty	0.65 (0.57, 0.74), <0.001	0.65 (0.57, 0.74), <0.001	0.69 (0.59, 0.81), <0.001
CoronaVac	0.70 (0.63, 0.78), <0.001	0.70 (0.63, 0.78), <0.001	0.70 (0.61, 0.79), <0.001
Multimorbidity status			
One chronic condition	-	Ref	Ref
Multimorbid	-	1.61 (1.47, 1.77), <0.001	1.65 (1.51, 1.80), <0.001
Interaction			
Comirnaty X multimorbidity	-	-	0.87 (0.67, 1.15), 0.332
CoronaVac X multimorbidity	-	-	1.03 (0.83, 1.27), 0.792

Included independent variable in Model 1: vaccine groups only; Model 2: Model 1 + multimorbidity status; Model 3: Model 2 + interaction between vaccination status and multimorbidity

Supplementary Table 14. Hazard ratios with 95% confidence intervals (CI) of adverse events of interest generated from Cox proportional hazard models with entropy rebalancing (1:3 matching with replacement)

	Hazard ratios (95% CI)		
	Model 1	Model 2	Model 3
Vaccine groups			
Unvaccinated	Ref	Ref	Ref
Comirnaty	0.64 (0.56, 0.72), <0.001	0.64 (0.57, 0.72), <0.001	0.65 (0.56, 0.76), <0.001
CoronaVac	0.50 (0.45, 0.55), <0.001	0.51 (0.46, 0.56), <0.001	0.48 (0.43, 0.55), <0.001
Multimorbidity status			
One chronic condition	-	Ref	Ref
Multimorbid	-	1.57 (1.48, 1.66), <0.001	1.56 (1.47, 1.65), <0.001
Interaction			
Comirnaty X multimorbidity	-	-	0.96 (0.74, 1.24), 0.749
CoronaVac X multimorbidity	-	-	1.11 (0.91, 1.36), 0.307

Included independent variable in Model 1: vaccine groups only; Model 2: Model 1 + multimorbidity status; Model 3: Model 2 + interaction between vaccination status and multimorbidity

Supplementary Table 15. List of included chronic conditions and corresponding diagnosis codes

Disease	ICD-9 codes	ICPC codes
Dementia	290, 294.1, 331.2	P70
Hypertension	401-405	K86, K87
Irritable bowel syndrome	564.1	D93
Peripheral vascular disease	440.2	-
Schizophrenia	295	P72
Alcohol misuse	265.2, 291.1–291.3, 291.5– 291.9, 303.0, 303.9, 305.0, 357.5, 425.5, 535.3, 571.0– 571.3, 980, V11.3	P15
Chronic pain	307.80, 307.89, 338.0, 338.2, 338.4, 719.41, 719.45 - 719.47, 719.49, 720.0, 720.2, 720.9, 721.0 - 721.4, 721.6, 721.8, 721.9, 722, 723.0, 723.1, 723.3 - 723.9, 724.0 - 724.6, 724.70, 724.79, 724.8, 724.9, 729.0 - 729.2, 729.4, 729.5	-
Depression	296.2, 296.3, 296.5, 300.4, 309, 311	P76
Hypothyroidism	240.9, 243, 244, 246.1, 246.8	T86
Parkinson's disease	332	N87
Psoriasis	696.1	S91
Severe constipation	560.1, 560.30, 560.39, 560.9, 564.0, 569.83, 569.89	D12
Asthma	493	R96
Chronic pulmonary disease	416.8, 416.9, 490–492, 494-505, 506.4, 508.1, 508.8	R95
Inflammatory bowel disease	555, 556	-
Peptic ulcer disease	440.2	D86
Rheumatoid arthritis	446.5, 710.0–710.4, 714.0– 714.2, 714.8, 725	L88
Diabetes (Type 2)	250.00, 250.02, 250.10, 250.12, 250.20, 250.22, 250.30, 250.32, 250.40, 250.42, 250.50, 250.52, 250.60, 250.62, 250.70, 250.72, 250.80, 250.82, 250.90, 250.92	-
Cancer	200–202, 203.0, 238.6, 196–199, 153-154, 162-163, 174, 180, 185, 230.3-230.6, 231.2, 233.0-233.1, 233.4	B72, B74, D74, D75, D76, D77, L71, N74, R84, S77, T71, U75, U76, U77, W72, X75, X76, X77, Y77, Y78
Cirrhosis	571.2, 571.5, 571.6, 456.0, 456.1, 456.20, 456.21, 567.0, 567.2, 567.21, 567.29, 567.8, 567.9, 572.2, 572.4, 789.5	-

Supplementary Table 16. List of adverse events of interest and corresponding diagnosis codes

Adverse events of interest	ICD-9 codes	ICPC codes
Auto-immune diseases		
Guillain-Barré Syndrome	357.0, 357.8x, 357,9	N94, N94005
Acute disseminated encephalomyelitis (ADEM)	323.6x, 323.8x	
Narcolepsy	347.xx, 89.17, 89.18, 307.4, 780.5	
Acute aseptic arthritis	274.0x, 696.0, 716.5x, 716.6x, 716.9x, 712.xx, 711.5x	
Type 1 Diabetes	250.01, 250.03, 250.11, 250.13, 250.21, 250.23, 250.31, 250.33, 250.41, 250.43, 250.51, 250.53, 250.61, 250.63, 250.71, 250.73, 250.81, 250.83, 250.91, 250.93	T89
(Idiopathic) Thrombocytopenia	287.3x, 287.4x, 287.5, 279.12, 283.11, 284.1x, 446.6, 776.1	
Subacute thyroiditis	245.1	
Cardiovascular system diseases		
Microangiopathy	446.6	
Heart failure	428.xx, 398.91, 402.01, 402.11, 402.91, 404.01, 404.03, 404.11, 404.13, 404.91, 404.93	K77
Stress cardiomyopathy	429.83	
Coronary artery disease	410.xx-414.xx, V45.81, 36.0x, 36.1x, 36.2x, 36.3x	K74, K75, K76
Arrhythmia	427.xx, 426.7, 624.x, 794.3x, 785.0	K79, K80
Myocarditis	422.xx, 429.0, 420.9, 423.9	K84, K84010
Circulatory system diseases		
Thromboembolism	415.1x, 453.xx, 443.xx, 444.xx, 445.xx, 433.xx, 434.xx, 435.xx, 436, 437.0, 437.1, 437.6, 437.8, 437.9, 451.x, 452.x, 325, 286.6x, 459.9, 434.01, 557.0, 557.9, 453.40, 453.41, 453.42, 410.x	K74, K75, K76, K89, K90, K91, K93, K94
Hemorrhagic disease	286.5x, 286.7, 287x, 430, 431, 432.0, 432.9	K90, K91
Single Organ Cutaneous Vasculitis	709.1, 446.2x, 287.0	B83, K99, B83019, K99016
Hepato-renal system diseases		
Acute liver injury	570.xx, 573.3	D80, D97
Acute kidney injury	584.xx, 586.xx	U99, U99005
Acute pancreatitis	577.0	
Nerves and central nervous system diseases		
Generalized convulsion	345.xx, 780.3x, 779.0, 333.2, 649.4	N07, N88

Meningoencephalitis	322.9x, 323.0x, 323.4x, 323.5x, 323.6x, 323.8x, 323.9x, 323.7, 330.8, 337.73, 046.3, 049.8, 036.1, 056.01, 136.2, 130, 054.3, 049.0, 094.1, 072.2, 013.0, 062.4, 049.9, 045.0, 062	N71, N70
Transverse myelitis	323.0x, 323.4x, 323.5x, 323.6x, 323.8x, 341.2x	
Bell's Palsy	351.0, 351.8, 351.9	N91
Respiratory system disease		
Acute respiratory distress syndrome	518.8x, 518.5x, 96.7x	R99, R99004
Skin, bone, and joints system diseases		
Erythema multiforme	695.1x	
Chilblain – like lesions	991.5	A88, A88001
Other system diseases		
Anosmia, ageusia	781.1	N16
Anaphylaxis	995.0, 995.1, 995.3, 995.4, 999.4x, 708.9, 519.11, 786.1, 458.9, 519.1x	A12, A99, A84, A85
Multisystem inflammatory syndrome in children	446.1	B99, B99022
Rhabdomyolysis	728.88, 728.89, 791.3	
