# Supplementary material for: Multimorbidity and adverse events of special interest associated with Covid-19 vaccines in Hong Kong
Source: Nat Commun. 2022 Jan 20;13:411. doi: 10.1038/s41467-022-28068-3 (PMC8776841; doi:10.1038/s41467-022-28068-3)
Supplement: Supplementary file 3 — Reporting Summary [file 41467_2022_28068_MOESM3_ESM.pdf]

nature portfolio

Corresponding author(s): Ian Chi Kei Wong

Last updated by author(s): Dec 20, 2021

Reporting Summary

Nature Portfolio wishes to improve the reproducibility of the work that we publish. This form provides structure for consistency and transparency in reporting. For further information on Nature Portfolio policies, see our Editorial Policies and the Editorial Policy Checklist.

Statistics

For all statistical analyses, confirm that the following items are present in the figure legend, table legend, main text, or Methods section.

n/a	Confirmed	
☐	☒	The exact sample size (*n*) for each experimental group/condition, given as a discrete number and unit of measurement
☒	☐	A statement on whether measurements were taken from distinct samples or whether the same sample was measured repeatedly
☐	☒	The statistical test(s) used AND whether they are one- or two-sided<br>*Only common tests should be described solely by name; describe more complex techniques in the Methods section.*
☐	☒	A description of all covariates tested
☐	☒	A description of any assumptions or corrections, such as tests of normality and adjustment for multiple comparisons
☐	☒	A full description of the statistical parameters including central tendency (e.g. means) or other basic estimates (e.g. regression coefficient) AND variation (e.g. standard deviation) or associated estimates of uncertainty (e.g. confidence intervals)
☐	☒	For null hypothesis testing, the test statistic (e.g. *F*, *t*, *r*) with confidence intervals, effect sizes, degrees of freedom and *P* value noted<br>*Give P values as exact values whenever suitable.*
☒	☐	For Bayesian analysis, information on the choice of priors and Markov chain Monte Carlo settings
☐	☒	For hierarchical and complex designs, identification of the appropriate level for tests and full reporting of outcomes
☒	☐	Estimates of effect sizes (e.g. Cohen's *d*, Pearson's *r*), indicating how they were calculated

Our web collection on statistics for biologists contains articles on many of the points above.

Software and code

Policy information about availability of computer code

Data collection	No specific software was used in the collection of data.
Data analysis	All analyses were conducted using the R statistical environment (Version 4.1.1, Vienna, Austria). For the weighting of the cohort, R package ‘WeightIt’ (Version 0.12.0) was used and ‘Survival’ (Version 3.2-13) was used for Cox regression. The code used for this study is available on Zenodo (https://doi.org/10.5281/zenodo.5792703).

For manuscripts utilizing custom algorithms or software that are central to the research but not yet described in published literature, software must be made available to editors and reviewers. We strongly encourage code deposition in a community repository (e.g. GitHub). See the Nature Portfolio guidelines for submitting code & software for further information.

Data

Policy information about availability of data

All manuscripts must include a data availability statement. This statement should provide the following information, where applicable:

- Accession codes, unique identifiers, or web links for publicly available datasets
- A description of any restrictions on data availability
- For clinical datasets or third party data, please ensure that the statement adheres to our policy

De-identified electronic medical records of patients were provided by the Hospital Authority, Hong Kong SAR, China. De-identified vaccination records were provided by the Department of Health, Hong Kong SAR, China. Data access is restricted to approved individuals through written agreements with the authors, subject to the approval by the data providers (Hospital Authority and Department of Health, Hong Kong SAR, China).

Field-specific reporting

Please select the one below that is the best fit for your research. If you are not sure, read the appropriate sections before making your selection.

☒ Life sciences ☐ Behavioural & social sciences ☐ Ecological, evolutionary & environmental sciences

For a reference copy of the document with all sections, see nature.com/documents/nr-reporting-summary-flat.pdf

Life sciences study design

All studies must disclose on these points even when the disclosure is negative.

Sample size	No sample size calculation was conducted because of the novel use of COVID-19 vaccines, it was not possible for this pharmacovigilance to predict the effect size. Methodologically, this is a population-based study and all identified eligible patients from the territory-wide healthcare database were included in the analysis.
Data exclusions	Part of the sample was excluded during the age- and sex-matching procedure. In addition, patients hospitalized on the index date, those having chronic disease diagnoses only after the index date, those who experienced the outcome event before the index date, and those who died before the index date were excluded.
Replication	A series of sensitivity analyses were conducted on the same study sample, with no substantial deviation from the main results observed.
Randomization	This is an observational study.
Blinding	This is an observational study.

Reporting for specific materials, systems and methods

We require information from authors about some types of materials, experimental systems and methods used in many studies. Here, indicate whether each material, system or method listed is relevant to your study. If you are not sure if a list item applies to your research, read the appropriate section before selecting a response.

Materials & experimental systems

n/a	Involved in the study	
☒	☐	Antibodies
☒	☐	Eukaryotic cell lines
☒	☐	Palaeontology and archaeology
☒	☐	Animals and other organisms
☐	☒	Human research participants
☒	☐	Clinical data
☒	☐	Dual use research of concern

Methods

n/a	Involved in the study	
☒	☐	ChIP-seq
☒	☐	Flow cytometry
☒	☐	MRI-based neuroimaging

Human research participants

Policy information about studies involving human research participants

Population characteristics	Patients who received inpatient or outpatient services provided by the Hospital Authority between January 1, 2018 and July 31, 2021, and ever coded with a diagnosis of any of 20 chronic conditions in their medical records since 2018 based on a widely used list of conditions for multimorbidity operationalization, including hypertension, diabetes mellitus (type 2), severe constipation, depression, cancer, hypothyroidism, chronic pain, asthma, alcohol misuse, chronic pulmonary disease, schizophrenia, rheumatoid arthritis, peptic ulcer disease, cirrhosis, psoriasis, Parkinson's disease, dementia, irritable bowel syndrome, inflammatory bowel disease, and peripheral vascular disease using International Classification of Diseases, Ninth Revision (ICD-9) and International Classification of Primary Care, Second Edition (ICPC-2).
Recruitment	De-identified electronic medical records of patients (aged 16 years or older) were provided by the Hospital Authority (HA), the sole provider of public inpatient services and a major provider of public outpatient services in Hong Kong. De-identified vaccination records were provided by the Department of Health were linked by matching a unique person ID between the two databases. We retrieved the records of patients who received inpatient or outpatient services provided by the HA during January 1, 2018 – July 31, 2021 and selected those ever coded with a diagnosis of any of 20 chronic conditions in the medical records since 2005 based on a widely used list of conditions for multimorbidity operationalization.
Ethics oversight	This study was approved by the institutional review board of the University of Hong Kong / Hospital Authority Hong Kong West Cluster (UW 21-149 and UW 21-138) and the Department of Health Ethics Committee (LM 21/2021).

Note that full information on the approval of the study protocol must also be provided in the manuscript.
